# Effects of Khat (*Catha edulis*) use on catalytic activities of major drug-metabolizing cytochrome P450 enzymes and implication of pharmacogenetic variations

**DOI:** 10.1038/s41598-018-31191-1

**Published:** 2018-08-24

**Authors:** Worku Bedada, Fernando de Andrés, Ephrem Engidawork, Jemal Hussein, Adrián LLerena, Eleni Aklillu

**Affiliations:** 1 Department of Pharmacology and Clinical Pharmacy, School of Pharmacy, College of Health Sciences, Addis Ababa University, P.O Box 1176, Addis Ababa, Ethiopia; 2 CICAB Clinical Research Centre, Extremadura University Hospital & Medical School, E-06071 Badajoz, Spain; 3 Department of Pharmacy, Jimma University, Jimma, Ethiopia; 4 Division of Clinical Pharmacology, Department of Laboratory Medicine, Karolinska Institutet, Karolinska University Hospital-Huddinge C1:68, SE-141 86 Stockholm, Sweden

## Abstract

In a one-way cross-over study, we investigated the effect of Khat, a natural amphetamine-like psychostimulant plant, on catalytic activities of five major drug-metabolizing cytochrome P450 (CYP) enzymes. After a one-week Khat abstinence, 63 Ethiopian male volunteers were phenotyped using cocktail probe drugs (caffeine, losartan, dextromethorphan, omeprazole). Phenotyping was repeated after a one-week daily use of 400 g fresh Khat leaves. Genotyping for *CYP1A2*, *CYP2C9*, *CYP2C19*, *CYP2D6*, *CYP3A5* were done. Urinary cathinone and phenylpropanolamine, and plasma probe drugs and metabolites concentrations were quantified using LC-MS/MS. Effect of Khat on enzyme activities was evaluated by comparing caffeine/paraxanthine (CYP1A2), losartan/losartan carboxylic acid (CYP2C9), omeprazole/5-hydroxyomeprazole (CYP2C19), dextromethorphan/dextrorphan (CYP2D6) and dextromethorphan/3-methoxymorphinan (CYP3A4) metabolic ratios (MR) before and after Khat use. Wilcoxon-matched-pair-test indicated a significant increase in median CYP2D6 MR (41%, p < 0.0001), and a marginal increase in CYP3A4 and CYP2C19 MR by Khat. Repeated measure ANOVA indicated the impact of *CYP1A2* and *CYP2C19* genotype on Khat-CYP enzyme interactions. The median MR increased by 35% in CYP1A2*1/*1 (p = 0.07) and by 40% in carriers of defective *CYP2C19* alleles (p = 0.03). Urinary log cathinone/phenylpropanolamine ratios significantly correlated with CYP2D6 genotype (p = 0.004) and CYP2D6 MR (P = 0.025). Khat significantly inhibits CYP2D6, marginally inhibits CYP3A4, and genotype-dependently inhibit CYP2C19 and CYP1A2 enzyme activities.

## Introduction

Khat (*Catha edulis* Forsk) is the most widely used psychoactive herb in the world^1,2^. Fresh leaves of Khat are used by millions of people as a recreational drug on daily bases for its euphoric and psychostimulant effect. Khat leaves are chewed slowly over several hours, and the juice of the masticated leaves is swallowed as part of deep-rooted socio-cultural tradition of the indigenous population living in East Africa and the Arabian Peninsula^1,3,4^, while the habit is spreading to Europe and the United States with the influx of migrants^5–7^. The chronic use of Khat causing psychological dependence has now become a growing public health problem not only in East Africa and Arabian Peninsula but also in Europe^8–10^.

Khat contains more than 40 alkaloids, but its stimulant effect derives mainly from cathinone, the main psychostimulant alkaloid in Khat, which is dubbed as “natural amphetamine” due to its structural and pharmacological similarity^1,11^. Cathinone undergoes a rapid Phase I stereo selective keto reduction by liver microsomal enzymes^12^ to norephedrine and cathine^13^, but the enzymes catalyzing this metabolism have not yet been elucidated. However, it is predicted from the metabolic pathways of amphetamines and synthetic cathinones that major cytochrome P450 (CYPs) might be involved^14^.

Cultivated commercially and freely available, Khat has been used for generations in Ethiopia by all walks of life, including children, pregnant, breastfeeding women and patients on medication^4,15–18^. For instance, Khat use is common among HIV patients, about 75% reporting lifetime use and 65% reporting use within the previous year^15^. The likelihood of Khat-drug interactions could be higher than drug-drug interactions, because drugs usually contain single chemical entities, while almost all herbs contain mixtures of pharmacologically active constituents^19–21^. Therefore, herb-drug interactions may pose a potential risk for patients on medication with narrow therapeutic range drugs to cause serious clinical consequences^19^.

The CYP450 enzymes are susceptible to inhibition or induction by natural products including herbal medicines that contain mixture of phytochemicals^21–23^. Before and after first-line phenotype screening studies using cocktail probe substrates approach focusing on five of the major CYP450 enzymes that metabolizes more than 90% of clinically used drugs, namely CYP1A2, 2C9, 2C19, 2D6, and 3A4, predict herb-drug interactions^24–26^. Despite the regular use of Khat by millions of people for centuries, little is known about the potential Khat-drug interactions. Few studies in Yemen reported that Khat chewing significantly reduced the bioavailability of ampicillin and chloroquine^27,28^ but the mechanism behind these observations remain unknown. In a small sample size preliminary study, we recently reported a significant inhibition of CYP2D6 and a borderline effect on CYP3A4 metabolic activity by Khat use^29^. The effect of Khat use on the other CYP enzymes and the impact of pharmacogenetic variations warrants further investigations. Consequently, this study was undertaken to evaluate the effect of Khat use on the metabolic activities of five major drug metabolizing CYP enzymes in humans (CYP1A2, CYP2C9, CYP2C19, CYP2D6, and CYP3A4), and the potential influence of pharmacogenetic variations on Khat-CYP enzymes interactions.

## Results

Sixty-three healthy unrelated Ethiopian male volunteers who were residents of Addis Ababa and regular Khat users were enrolled and participated in the phase I study, but four participants missed the phase II study. Most of the study participants were undergraduate students at Addis Ababa University with more than three years of Khat chewing experiences (median: 6 years), with a frequency of chewing Khat at least three days per week (median, 5 days per week). The median age of the study participants was 25 years old (IQR 24–34 years). Forty-six (73%) of them were cigarette smokers, and three individuals (5%) reported to inhale Shisha (water pipe tobacco smoking). After enrolment, the participants were instructed to abstain from Khat chewing for one week for baseline assessment of CYP enzymes activity. Based on the reported short half-life of cathinone (1.5 ± 0.8 h) and cathine (5.2 ± 3.4 h)^30^, a one week washout period was considered to be sufficient to eliminate cathinone and cathine from previous Khat ingestion. Furthermore, cathinone and cathine are detectable in urine only up to 22–26 h and 50–70 h respectively after ingestion^31^.

Khat withdrawal symptoms were observed in almost all study participants after one week of Khat abstinence according to the DSM-V criteria for stimulant withdrawal^32^. Most participants experienced dysphoria, fatigue, and increased appetite, and agitation was observed in 70% of the subjects. Moreover, most of the participants reported hypersomnia, vivid and unpleasant dreams. Traces of urinary cathinone were detected in 9 subjects from phase 1 sampling, and data were analyzed by both including and excluding these subjects.

**Figure 1:**
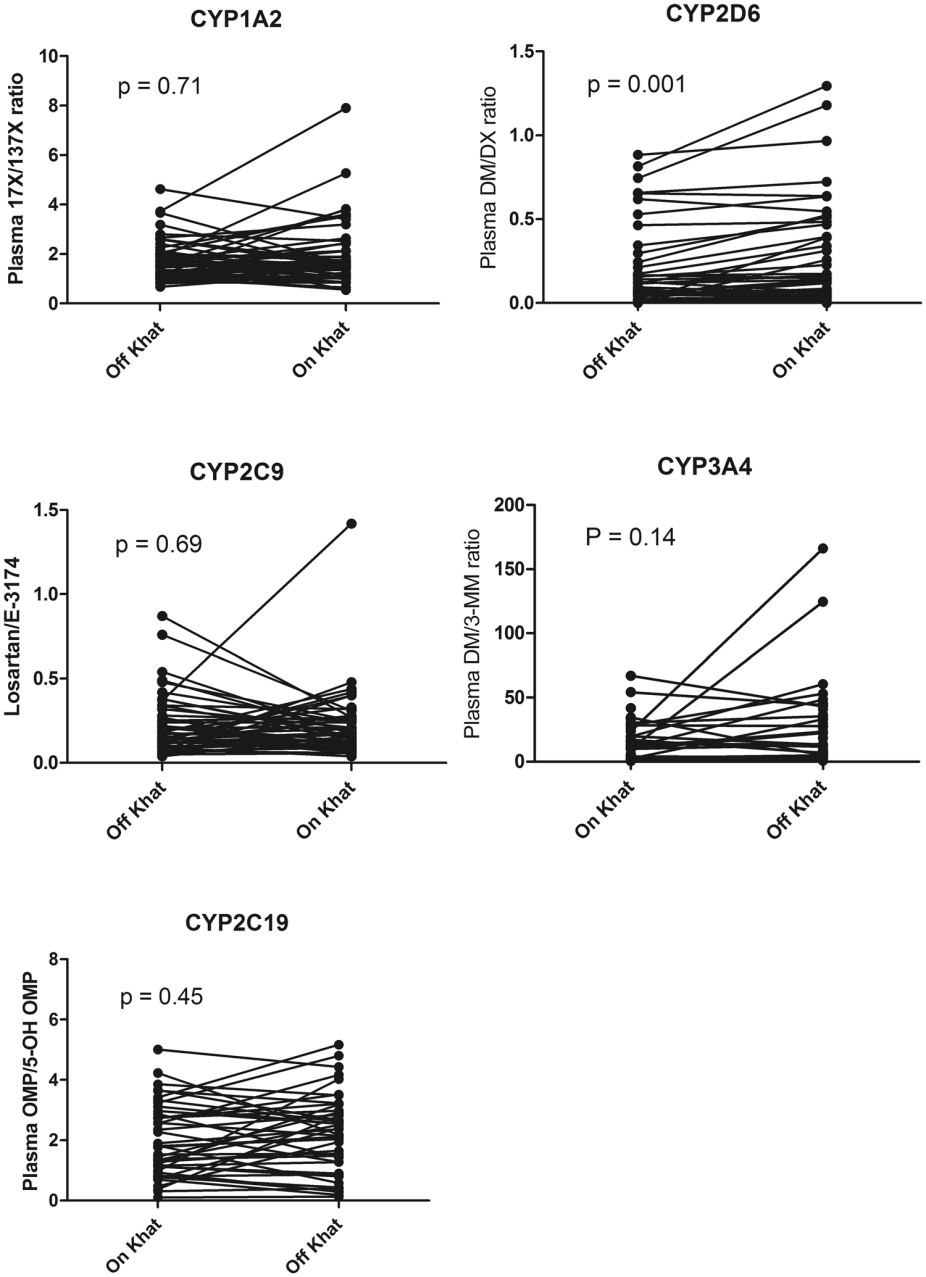
Comparisons of plasma CYPs metabolic ratios before and after Khat consumption among extensive metabolizers of CYP1A2, CYP2C9, CYP2C19, CYP2D6 and CYP3A4 using Wilcoxon matched-pairs signed rank test.

Wilcoxon matched pair test, and the respective median MR percent change from baseline in the presence of Khat is presented in Table 1. A case profile line plot indicating within-subject change in CYP MRs among extensive metabolizers in the absence and presence of Khat is presented in Fig. 1. Observed genotype and allele frequencies are presented in Table 2. There were no significant differences between the observed and expected genotype frequencies according to Hardy–Weinberg equilibrium. Comparison of median and interquartile range of CYP MRs in the absence of Khat (baseline) and after one week of Khat use along with the respective median percent change from baseline stratified genotype is presented in Table 3.

**Table 1:** Comparisons of median MR and IQR obtained for CYP1A2, CYP2C9, CYP2C19, CYP2D6 and CYP3A4 in the absence of Khat and after its consumption.

Enzyme	Metabolic Ratio	Off Khat Median (IQR)	On Khat Median (IQR)	Median percent change	P-value (Wilcoxon matched pair test)
*CYP1A2*	Caffeine/paraxanthine 0–4 h	1.64 (1.19–1.99)	1.52 (1.10–2.11)	−3.1%	0.70
*CYP2C9*	Losartan/losartan carboxylic acid 0–4 h	0.16 (0.09–0.34)	0.18 (0.11–0.27)	1.1%	0.55
*CYP2C19*	Omeprazole/5-hydroxyomeprazole 0–4 h	1.90 (1.07–3.24)	2.37 (1.28–3.21)	10.4%	0.15
*CYP2D6*	Dextromethorphan/dextrorphan 0–3 h	0.08 (0.03–0.27)	0.12 (0.04–0.47)	41.2%	0.002
*CYP3A4*	Dextromethorphan/3-methoxymorphinan 0–3 h	11.7 (4.0–26.2)	18.5 (6.9–33.5)	30.5%	0.13

IQR = Interquartile range.

**Table 2:** Genotype and alleles frequency within the population studied. CYP2D6*3 and CYP3A5*7 were not detected.

CYP enzyme	Genotype	Frequency % (n)
*CYP1A2*	*1/*1	20.6% (13)
	*1/*1F	42.9% (27)
	*1F/*1F	36.5% (23)
*CYP2C9*	*1/*1	77.8% (49)
	*1/*2	14.3% (9)
	*1/*3	(6.3%) (4)
	*2/*2	1.6% (1)
*CYP2C19*	*1/*1	79.4% (50)
	*1/*2	19.0% (12)
	*1/*3	1.6% (1)
*CYP2D6*	*1/*1	84.1% (53)
	*1/*4	14.3% (9)
	*4/*4	1.6% (1)
*CYP3A5 c*.*6986A* > G (*3)	*1/*1	23.8% (15)
	*1/*3	49.2% (31)
	*3/*3	27.0% (17)
*CYP3A5 c*.*14690G* > A (*6)	*1/*1	68.3% (43)
	*1/*6	28.6% (18)
	*6/*6	3.2% (2)
Number of CYP3A5*1 alleles^a^	Zero	19 (30.2%)
	One	37 (58.7%)
	Two	7 (11.1%)
**Gene**	**Allele**	**Frequency (%)**
*CYP1A2*	*−163C* > A (1A2*1F)	52.1
*CYP2C9*	*c*. 430*C* > T (2C9*2)	8.7
	*c*.*1075A* > C (2C9*3)	3.2
*CYP2C19*	*c*. 681*G* *>* A (2C19*2)	9.5
	*c*. 636*G* > A (2C19*3)	0.8
*CYP2D6*	*c*.*2549delA* (2D6*3)	ND
	*c*. *1846G* > A (2D6*4)	8.7
*CYP3A5*	*c*.*6986A* > G (3A5*3)	51.6
	*c*.*14690G* > A (3A5*6)	17.5
	*c*.*27131_27132insT* (3A5*7)	ND

^a^CYP3A5*1 = absence of CYP3A5*3, CYP3A5*6 and/or CYP3A5*7, ND = not detected.

**Table 3:** Median and inter quartile range of CYP metabolic ratios (MR) obtained in the absence and after consumption of Khat, and the respective percent change in MR from baseline calculated among the different genotype groups.

CYP enzyme	Metabolic Ratio	Genotype	n	Without Khat	n	With Khat	Median % change from baseline	P-value^a^	P-value^b^
CYP1A2	Caffeine/paraxanthine 0–4 h	*1/*1	13	1.52 (1.19–1.98)	12	1.65 (1.46–3.08)	35.2	0.07	0.04
		*1/*1F	27	1.63 (1.09–1.98)	26	1.43 (1.04–1.75)	−5.8	0.31	
		*1F/*1F	23	1.73 (1.44–1.99)	21	1.66 (1.07–2.13)	−1.9	0.84	
CYP2C9	Losartan/losartan carboxylic acid 0–4 h	*1/*1	49	0.15 (0.09–0.34)	46	0.16 (0.11–0.26)	−5.5	0.20	0.31
		*1/*2 or *3	13	0.18 (0.12–0.28)	12	0.22 (0.15–0.29)	−0.3	0.42	
		*2/*2	1	1.06	1	1.46	38		
CYP2C19	Omeprazole/5-hydroxyomeprazole 0–4 h	*1/*1	50	1.82 (0.98–3.12)	47	2.12 (0.9–2.82)	7.1	0.83	0.02
		*1/*2 or *3	13	2.73 (1.07–3.64)	12	3.20 (2.98–3.52)	40.3	0.03	
CYP2D6	Dextromethorphan/dextrorphan 0–3 h	*1/*1	53	0.06 (0.02–0.16)	50	0.10 (0.04–0.29)	40.1	0.003	0.06
		*1/*4	9	0.66 (0.17–3.21)	8	0.72 (0.06–1.56)	9.98	0.34	
		*4/*4	1	34.38	1	37.5	9.13		
No of CYP3A5*1 allele	Dextromethorphan/3-methoxymorphinan 0–3 h	3A5 *wt/wt*	7	9.63 (5.06–40.29)	6	13.44 (10.99–23.86)	9.60	0.65	0.13
		heterozygous	37	12.08 (2.97–19.91)	35	15.94 (5.69–33.09)	30.2	0.07	
		mut/mut^c^	19	10.17 (4.01–33.6)	18	15.67 (4.89–35.50)	19.5	0.57	

n: number of individuals.

^a^P-value from Wilcoxon Matched Pairs Test.

^b^Repeated measure ANOVA^a^ using log transformed metabolic ratios.

^c^CYP3A5*3 or *6.

### Effect of Khat on CYP1A2 enzyme activity

CYP1A2 activity was determined using plasma caffeine/paraxanthine ratio. There were no significant differences in the median CYP1A2 MRs in the absence or presence of Khat regardless of smoking habit (Table 1), Paired t-test indicated no significant differences in the mean log CYP1A2 MR determined before and after Khat (p = 0.89, geometric mean ratio (GMR) = 0.991; 95% CI of GMR (0.864 to 1.135). No significant effect of CYP1A2*1F genotype on log CYP1A2 MR irrespective of Khat use was found. Repeated measure ANOVA indicated a significant interaction between *CYP1A2* genotype and variations in CYP1A2 MR by Khat, with an increased MR from baseline in CYP1A2*1/*1 genotypes, but not in CYP1A2*1F carriers (Table 3). However, though not significant, a median 35% increase in CYP1A2 MR from baseline in the presence of Khat was observed among CYP1A2*1/*1 genotypes (*p* = 0.07). Smokers had lower log CYP1A2 MR than non-smokers at baseline (*p* = 0.15) and in the presence of Khat (*p* = 0.05). A non-significant increase (10%) in the median percent change of CYP1A2 MR from baseline was observed in non-smokers but not change in smokers was observed.

### Effect of Khat on CYP2C9 enzyme activity

CYP2C9 activity was determined using plasma losartan/losartan carboxylic acid ratio (CYP2C9 MR). Three subjects (4.8%) were 2C9 poor metabolizers (PMs) with plasma losartan/losartan carboxylic acid ratio >1 at baseline. Only one of the PMs was homozygous 2C9*2/*2, but the rest were *1/*1 genotype. Paired t-test indicated no significant differences in the mean log CYP2C9 MR determined before and after Khat (p = 0.75, GMR = 1.038; 95% CI of GMR = 0.864 to 1.309). *CYP2C9* genotype influenced the CYP2C9 MR in the presence (*p* = 0.06) or absence of Khat (*p* = 0.01). There was no significant change in the median CYP2C9 MR before and after Khat use by the participants, whereas the repeated measure ANOVA indicated no significant effect of *CYP2C9* genotype on within-subject variation of log CYP2C9 MR before and after Khat use.

### Effect of Khat on CYP2C19 enzyme activity

CYP2C19 activity was determined using plasma omeprazole/5-hydroxyomeprazole ratio (CYP2C19 MR). Three subjects (4.8%) had CYP2C19 MR > 0.8 in the absence of Khat and were assigned as CYP2C19 PMs, even though only one of them was heterozygous 2C19*1/*2 and the others were *1/*1. A non-significant increase (10%) in the median percent change of MRs from baseline was observed by Khat use. Influence of *CYP2C19* genotype on CYP2C19 MR at baseline (*p* = 0.07) and after one week Khat use (*p* = 0.01) was observed. There was no significant differences in the mean log CYP2C19 MRs determined before and after Khat (p = 0.49, GMR = 0.934; 95% CI of GMR = 0.763 to 1.143). Repeated measure ANOVA indicated a significant effect of *CYP2C19* genotype on within subject variation of log CYP2C19 MR before and after Khat use (*p* = 0.02). Moreover, the median CYP2C19 MR percent increase from baseline by Khat was significantly higher in carriers of CYP2C19*2 or *3 (40%) compared to *1/*1 genotypes (7%).

### Effect of Khat on CYP2D6 enzyme activity

CYP2D6 enzyme activity was determined using plasma dextromethorphan/dextrorphan ratio (CYP2D6 MR). CYP2D6*3 was not detected. Two subjects with CYP2D6*4/*4 and *1/*4 genotype had log MR > 0.8 in the absence of Khat and were assigned as CYP2D6 PMs (3.2%). Their respective CYP2D6 MR increased from 34 to 37.5 and from 4.7 to 9.5 by Khat use, whereas another subject with CYP2D6*1/*4 genotype and a CYP2D6 MR of 0.67 at baseline became a poor metabolizer after one week of Khat use (MR = 1.56). Therefore, the prevalence of CYP2D6 PMs increased from 3.2% to 5.1% after Khat consumption.

Paired t-test indicated a significantly higher mean log CYP2D6 MR determined after a one week Khat use than before (p = 0.001, GMR = 0.758; 95% CI of GMR = 0.673 to 0.854).Considering all subjects, Wilcoxon matched-pair signed-rank test indicated a significant increase in CYP2D6 MR during Khat use (*p* = 0.002). More indeed, when only those subjects who were fully compliant to Khat abstinence were considered, the median CYP2D6 MR was strongly increased by Khat use compared to the baseline (Wilcoxon matched-pair signed-rank test, *p* = 0.001). The median percent change in CYP2D6 MR from baseline by Khat consumption was 41%. There was a significant effect of *CYP2D6* genotype on CYP2D6 MR regardless of Khat use. The median CYP2D6 MR increased by Khat was more pronounced in individuals with CYP2D6*1/*1 genotypes (40%) than in carriers of CYP2D6*4 (10%).

### Effect of Khat on CYP3A4 enzyme activity

CYP3A4 enzyme activity was determined using plasma dextromethorphan/3-methoxy-morphinan ratio (CYP3A4 MR). Compared to the baseline value, Wilcoxon matched-pair test indicated a non-significant increase in the median DM/3-MM ratio (*p* = 0.18), whereas a significant effect of *CYP3A5* genotype on MR regardless of Khat use was observed. Paired t-test indicated no significant differences between the mean log CYP3A4 MRs determined before and after Khat (p = 0.14, GMR = 0.706; 95% CI of GMR = 0.441 to 1.130). Repeated measure ANOVA indicated a marginal effect of *CYP3A5* genotype on the change in log DM/3 MM ratio MR during Khat consumption (*p* = 0.13). The increase in DM/3 MM ratio in the presence of Khat was higher in carriers of defective (CYP3A5*3 or *6) variant alleles (p = 0.07, paired t-test) but not in carriers of *1/*1 genotypes. CYP3A5*7 was not detected.

### Correlation between urinary cathinone/phenylpropanolamine ratio with CYP metabolic ratios and genotypes

**Figure 2:**
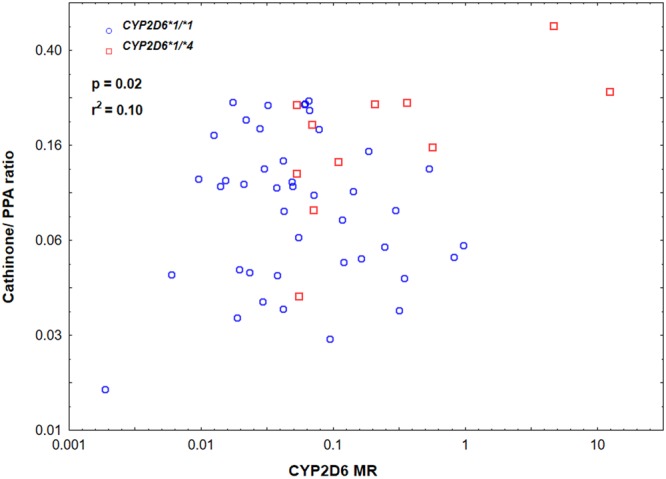
A scatter plot showing the correlation between plasma CYP2D6 metabolic ratio (MR) and urinary log cathinone/phenylpropanolamine (PPA) ratio stratified by CYP2D6 genotype.

Correlation of urinary log cathinone/phenylpropanolamine ratio with the MR of the different CYP enzymes and the influence of genotype on log cathinone/phenylpropanolamine ratio were analyzed. There was a positive significant correlation of log cathinone/ phenylpropanolamine ratio with CYP2D6 MR (*p* = 0.02; r^2^ = 0.10) as well as *CYP2D6* genotype (ANOVA, *p* = 0.004, F = 8.86). A scatter plot showing the correlation between plasma CYP2D6 MR and urinary log cathinone/ phenylpropanolamine ratios among the different CYP2D6 genotypes is presented in Fig. 2. On the contrary, no correlation between urinary log cathinone/phenylpropanolamine ratio and genotypes or metabolic ratios of CYP1A2, CYP2C9, CYP2C19, and CYP3A4 were observed.

## Discussion

In the present study, we investigated the effect of Khat use on the metabolic activities of five major drug metabolizing CYP enzymes and any potential influence of pharmacogenetic variations on the Khat-CYP enzymes interaction. In this one-way crossover study, each subject effectively functioned as its own control. Urinary cathinone and phenylpropanolamine were measured to monitor study participants’ compliance for Khat abstinence (for assessment of baseline CYP enzyme activities) and Khat intake (to determine Khat-CYP enzyme interaction) respectively. Our results indicate a significant Khat inhibitory effect on CYP2D6 (41% increment in the median MR percent change from baseline), and a marginal effect on CYP3A4 and CYP2C19 activities. The extent of enzyme inhibition by Khat was influenced by the respective genotype, except for CYP2C9. Thus, the inhibition of CYP2D6 by Khat was more pronounced in CYP2D6*1/*1 carriers than in individuals with CYP2D6*1/*4. Genotype-dependent inhibition of CYP2C19 and CYP1A2 by Khat was also observed. Khat use significantly inhibited CYP2C19 enzyme in subjects with reduced CYP2C19 enzyme activity (i.e., carriers of CYP2C19*defective alleles). On the other hand, CYP1A2 enzyme activity was significantly reduced by Khat use in CYP1A2*1/*1 genotypes when compared to carriers of CYP1A2*1F.

Ethiopians display unique pharmacogenetic characteristics concerning CYP enzymes^33–36^. About one-third of the Ethiopians carry functionally active duplicated or multi-duplicated *CYP2D6* genes^33^. As CYP2D6 is not inducible, selection of multiple copies of active *CYP2D6* alleles may indicate adaptation to some environmental influence, including dietary or non-dietary sources. The geographic overlap between the occurrence of higher frequency of CYP2D6 gene duplication among populations living in Khat belt countries (Horn of Africa and Arabian Peninsula) is noteworthy. Interestingly, having the same *CYP2D6* genotypes, Ethiopians living in Ethiopia display lower CYP2D6 enzyme activity when compared to those living in Sweden^14^, indicating the relevance of environmental factor in regulating CYP2D6 enzyme activity. Khat is rich in alkaloids, to which CYP2D6 enzyme has a high affinity. We postulated that Khat which is frequently used in Ethiopia but nearly absent in Sweden might explain the lower CYP2D6 enzyme activity in Ethiopians living in Ethiopia compared to those in Sweden. In line with our hypothesis and replicating our previous preliminary finding^29^, the present study further confirms a significant CYP2D6 enzyme inhibition by Khat use.

Cathinone undergoes stereospecific metabolism by liver microsomal enzymes to produce the phenylpropanolamine stereoisomers, cathine and norephedrine^13^, but the specific enzyme catalyzing this process is not elucidated. Interestingly, we found a significant correlation of log cathinone/phenylpropanolamine ratio, not only with CYP2D6 MR but also with *CYP2D6* genotype. Although identification and quantification of Khat constituents is beyond the scope of this study, CYP2D6 genotype-phenotype correlation with cathinone/phenylpropanolamine ratio may further implicate cathinone as a possible substrate and inhibitor of CYP2D6, and the potential role of CYP2D6 in the metabolism of cathinone to phenylpropanolamine. Accordingly, CYP2D6 enzyme inhibition by Khat might be due to competitive inhibition by cathinone.

The marginal effect of Khat on CYP3A4 and CYP2C19 enzyme activity shall not be underestimated, given the role of these enzymes in the metabolism of more than 50% of clinically used medications. Considering pharmacogenetic variations, an inhibitory effect of Khat was observed in carriers of defective variant alleles of *CYP2C19* (*2 or *3) and *CYP3A5* (*3 or *6), but not in subjects with *1/*1 genotype for these enzymes. Thus, subjects with low CYP2C19 or CYP3A4/5 enzyme activity are potentially at a higher risk for Khat-drug interaction. Our finding indicates that herb-drug interactions can be modified by pharmacogenetic variations affecting enzymes’ expression and/or their activity. However, no significant Khat effect on CYP2C9 enzyme activity was found. An additional interesting result from this study indicates a unique distribution of *CYP2C9* alleles in Ethiopians compared to Whites and Asians^37,38^. The allele frequency of CYP2C9*2 in Ethiopians (8.7%) is similar to Swedes (10.8%), whereas it is absent in Koreans. Additionally, the frequency of CYP2C9*3 in Ethiopians is much lower (3.2%) than in Swedes (12.5%) and Koreans (5.8%)^39^.

The present study has clinical implications. Chronic use of Khat is associated with a variety of mental and personality disorders that require treatment^40–44^, and CYP2D6 metabolizes several psychoactive drugs including psychotropic, anti-depressants and antipsychotics. Considerable inhibition of CYP2D6 by Khat use may result in unanticipated adverse events and/or treatment failures. CYP2D6 is constitutively expressed in human brain, where it is involved in endogenous metabolism including dopamine and serotonin^45,46^. Cathinone increases the levels of dopamine in the brain, possibly by acting on the cathecholaminergic synapses^47,48^. A previous clinical study suggested that CYP2D6 slow metabolizers might have a higher dopamine tone in the pituitary^49^, and the inhibition of CYP2D6 activity in the brain may be another mechanism by which cathinone, the main psychostimulant alkaloid in Khat, increases the levels of dopamine in the brain.

Concomitant Khat use while on medication is common in Ethiopia. A high regular Khat use while on antiretroviral therapy is well documented^8,18,50^. Moreover, *P*.*vivax* malaria is endemic in Ethiopia, and the only remedies chloroquine and primaquine are metabolized by CYP2D6. Indeed, the primaquine’s metabolite, which is responsible for hypnozoite killing, is also generated by CYP2D6^51^. A recent study by Issa *et al*., in Yemen reported that Khat-chewing significantly reduces plasma chloroquine concentrations in malaria patients^28^. However, the authors did not control for the amount of Khat used and the timing of the Khat-chewing sessions in relation to chloroquine administration. At therapeutic concentration, chloroquine is metabolized into desethylchloroquine primarily by CYP2C8 (60%) followed by CYP3A4 (25%), and CYP2D6 is a high affinity but a significantly low capacity enzyme to metabolize chloroquine^52^. Furthermore, a previous study reported no significant inhibition of CYP2D6 by chloroquine in human^53^. Thus Khat-chloroquine interaction reported by Issa *et* *al*., may not be at the CYP2D6 level. Nevertheless, concomitant Khat use may compromise the antimalarial activity of primaquine. In deed a significant association of low-activity CYP2D6 phenotypes with the initial relapse and number of malaria relapses is reported^51^. Therefore, Khat abstinence while on treatment with CYP2D6 substrate drugs is advisable.

In East Africa including Ethiopia, Khat is typically chewed by groups of men in a cultural and social gatherings. Traditionally, Khat consumption by women is considered socially unacceptable and rarely practiced openly. In Ethiopia Females were 77% less likely to chew khat as compared to males^54^. In the present study we intended to enroll regular Khat users of both sex, but were not able to get female volunteers, partly due to the social stigma attached to the practice. Involving only male participants but not females can be considered as limitation of the study.

## Conclusion

Results of this study indicates that Khat use significantly inhibit CYP2D6, marginally inhibit CYP3A4, and genotype-dependently inhibit CYP2C19 and CYP1A2 enzyme activities. Correlation of urinary cathinone/phenylpropanolamine ratio with CYP2D6 genotype and phenotype may implicate cathinone as a substrate and inhibitor of CYP2D6, and a potential role of CYP2D6 in the conversion of cathinone to phenylpropanolamine. Consequently, the mechanism of CYP2D6 enzyme inhibition by Khat could be due to competitive inhibition by cathinone. Significant Khat-CYP2D6 substrate drug interaction may cause unanticipated pharmacological consequences. Given the fact that regular Khat use while on medication is quite common in East Africa and Arabian Peninsula, Khat abstinence while on medication with CYP2D6 substrate drug is advisable. Future clinical studies are needed to investigate the impact of Khat-CYP enzyme interaction on treatment outcome including safety and efficacy.

## Methods

### Study participants

Healthy unrelated regular Khat users were recruited and enrolled in Addis Ababa, Ethiopia. Before study enrolment, all subjects were examined to be healthy at the Black Lion Specialized Hospital, Addis Ababa University. At study enrolment, socio demographic information including habits of coffee drinking, smoking of cigarette or shisha, use of alcohol and herbal medicines was collected using a detailed questioner. The study inclusion criteria were i), age more than 18 years ii) regularly use of Khat for more than two years, with a consumption frequency of at least 3 days per week, ii) willingness to refrain from using Khat for one week for the baseline assessment of CYP enzyme activity, iii) willingness to refrain from taking any caffeine-containing beverage such as coffee, tea, Coca-Cola, chocolate for at least 72 hr prior to CYP phenotyping procedure. Study participants were instructed to refrain from taking any medication including herbal medicines during the study period, and from consuming caffeine 72 hours before CYP phenotyping.

The study was performed as per the Declaration of Helsinki for human experimental research. All participants gave written informed consent, and ethical approval to conduct the study was obtained from the institutional review board of the College of Health Sciences, Addis Ababa University, and from the National Research Ethics Committee, Federal Ministry of Science and Technology, Ethiopia.

### Study design

Comparative, open-label, one-way crossover observational study was carried out in two phases to evaluate the effects of Khat consumption on the metabolic activities of CYP1A2, CYP2C9, CYP2C19, CYP2D6 and CYP3A using a cocktail of the following probe drugs caffeine, losartan, omeprazole and dextromethorphan.

In phase-1, subjects were requested to abstain from chewing Khat for one week as a wash out period for any prior Khat use. On day 8, subjects received 100 mg caffeine, 50 mg losartan, 20 mg omeprazole and 30 mg dextromethorphan orally. Blood samples were collected 3 h post-dose for determination of baseline (in the absence of Khat) metabolic activities of CYP2D6 and CYP3A4, and 4 h post-dose for CYP1A2, CYP2C9, CYP2C19)^55^. Khat withdrawal symptoms were assessed using the criteria set by DSM-V (Diagnostic and Statistical Manual of Mental Disorders, Fifth Edition) by a substance abuse specialist from St. Paul Specialized Hospital, Ethiopia.

In phase 2, subjects used 400 g of locally harvested fresh Khat leaves daily for one week. On day 16, CYP1A2, CYP2C9, CYP2C19, CYP2D6 and CYP3A phenotyping were repeated in the presence of Khat. Subjects used Khat until the last minute of blood sample collection. To monitor subjects’ compliance for Khat abstinence (in phase 1) and Khat intake (in phase 2), 10-mL urine samples were collected from all study participants just before administration of probe drugs on day 8 and day 16, respectively. Blood samples were centrifuged for 10 minutes at 3500 *x g* and plasma aliquots were stored at −20 °C until analysis. Blood sample was collected in EDTA containing vacutainer tube for CYP genotyping.

### Quantification of plasma drugs and metabolites

Quantification of plasma probe drugs and their respective metabolites were done using procedures described elsewhere^56^. In brief, plasma samples were first incubated with β-glucuronidase (pH 5) at 37 °C for 18 h to hydrolyse metabolite conjugates. Then, 200 µL of cold methanol and 200 µL of acetonitrile were added to precipitate plasma proteins. The sample was centrifuged and the supernatant was then evaporated at 40 °C under a stream of nitrogen and the dried extract was reconstituted in 500 µL of potassium dihydrogen phosphate buffer at pH 7.5. The extract was subjected to a solid phase extraction process, and the elution fraction was evaporated to dryness at 40 °C under a stream of nitrogen. Then, the dried extract was reconstituted in the mobile phase, and ten µL of the extract was injected onto the chromatographic system (Agilent 1200 Series HPLC system, Agilent, Santa Clara, CA).

Chromatographic separation was achieved by gradient elution at a flow rate of 0.4 mL min^−1^ and at a temperature of 30 °C, using a PoroshellSB-C18 column (75 mm × 3 mm internal diameter, 2.7 μm, from Agilent, Torrance, CA). Mobile phase consisted of 0.1% formic acid in water and 0.1% formic acid in acetonitrile, and all analytes were detected by tandem mass spectrometry (API2000 triple quadrupole mass spectrometer from AB Sciex, MA, USA) in selected reaction monitoring (SRM) mode, with positive electrospray ionization (ESI) for all analytes except for losartan carboxylic acid (E-3174), which was detected with negative electrospray ionization mode.

### Quantification of urinary cathinone and cathine concentration

Urinary cathinone and cathine concentration was determined as described previously^57^ using LC–MS/MS system consisted of a Waters Acquity UPLC (ultra-performance liquid chromatography) with a vacuum degasser, binary pumps, autosampler (12 °C) and sample manager connected to a Xevo TQ tandem mass spectrometer with MassLynx™/Target Lynx™ Software version 4.1 (Waters Co., Milford, MA, USA). In brief, a 50 μL aliquot of urine was added to an autosampler vial with 200 μL of IS working solution (40 ng). The vials were capped, vortexed for ~10 sec, and loaded onto the sample manager kept at 4 °C. Amphetamine, MDMA and metamphetamine (LGC Standards) in 100 µg mL^−1^ concentration were diluted to 500 ng mL^−1^ in double-distilled water and used as internal standards. The reference standards, cathinone-HCl and Phenylpropanolamine-HCl in 1 mg mL^−1^ ampoules (LGC Standards) were diluted in 0.1% formic acid to give 100 mg mL^−1^ solutions. Quality control (QC, 100 ng mL^−1^) and standards for calibration curves covering a 0–10000 ng mL^−1^ concentration range were prepared by dilution of working solutions of the analytes with blank urine. Lower limits of detection for cathinone and phenylpropanolamine was 100 ng mL^−1^ and 300 ng mL^−1^ respectively.

### CYP genotyping

Genomic DNA was isolated from peripheral blood leukocytes using QIAamp DNA Maxi Kit (QIAGEN GmbH, Hilden, Germany). Genotyping for the common defective variant alleles of the different CYP genes were done. Allelic discrimination reactions were performed using TaqMan® (Applied Biosystems, CA, USA) genotyping assays with the following ID number for each SNP: C—8881221_40 for CYP1A2*1F (rs762551), C—25625805_10 for CYP2C9*2 (rs1799853), C—27104892_10 for CYP2C9*3 (rs1057910), C—25986767_70 for CYP2C19*2 (rs4244285), C—27861809_10 for CYP2C19*3 (rs4986893), C—32407232_50 for CYP2D6*3 (rs35742686), C—27102431_D0 for CYP2D6*4 (rs3892097), C—26201809_30 for CYP3A5*3 (rs776746), C—30203950_10 for CYP3A5*6 (14690 G > A g.14690 G > A) and C—32287188_10 for CYP3A5*7 (rs241303343), as described previously^36,58,59^. Genotyping was performed using Quant Studio 12 K Flex Real-Time PCR system (Life Technologies Holding, Singapore, Singapore). The final volume for each PCR reaction was 10 μL, consisting of TaqMan fast advanced master mix (Applied Biosystems, Waltham, MA, USA), TaqMan 20X drug metabolism genotyping assays mix (Applied Biosystems) and 10 ng genomic DNA. Each genotype analysis was done using sequenced confirmed DNA samples as a control.

### Statistical Analysis

Chi-square test was used to compare the observed and expected allele frequencies according to the Hardy-Weinberg equilibrium. The change in the median metabolic ratios (MR) of caffeine/paraxanthine (CYP1A2 MR), omeprazole/5-hydroxyomeprazole (CYP2C19 MR), losartan/ losartan carboxylic acid (CYP2C9 MR), dextromethorphan/dextrorphan ratio (CYP2D6 MR) and dextromethorphan/3-methoxymorphinan ratio (CYP3A4 MR) in the presence and absence of Khat was analyzed using Wilcoxon matched-pairs signed rank test. Spearman coefficient was used to assess correlations between log MR of the different CYPs and log cathinone/phenylpropanolamine ratios.

Plasma metabolic ratios were transformed into log_10_ values for parametric statistical analysis, and the Shapiro-Wilk test for normality was applied. Paired t-test was used on log transformed metabolic ratios to evaluate the variation in the mean MR in the absence and presence of Khat. Percent change in MR from baseline, in MR in the presence of Khat was calculated using the following equation:

$$\begin{array}{c} \% \,change\,in\,MR\,=\,[\frac{MR\,in\,the\,presence\,of\,Khat-MR\,in\,the\,absence\,of\,Khat}{MR\,in\,the\,absence\,of\,Khat}]\times 100\end{array}$$

One-way repeated measure ANOVA was used to evaluate the effect of genotype on MRs calculated in the absence and presence of Khat. Graphical representation and statistical analyses were performed using GraphPad Prism 6 (GraphPad Software Inc., USA) and SPSS Statistics (IBM Corporation, Somers, NY) software, version 24.0 respectively. *P* values < 0.05 were considered to be statistically significant.
